# Editorial: A year in review: discussions in nuclear medicine

**DOI:** 10.3389/fmed.2024.1370082

**Published:** 2024-02-06

**Authors:** Domenico Albano, Giorgio Treglia

**Affiliations:** ^1^Division of Nuclear Medicine, Università degli Studi di Brescia and ASST Spedali Civili di Brescia, Brescia, Italy; ^2^Division of Nuclear Medicine, Ente Ospedaliero Cantonale, Imaging Institute of Southern Switzerland, Bellinzona, Switzerland; ^3^Faculty of Biomedical Sciences, Università della Svizzera italiana, Lugano, Switzerland; ^4^Faculty of Biology and Medicine, University of Lausanne, Lausanne, Switzerland

**Keywords:** nuclear medicine, imaging, PET, oncology, theranostics, COVID, cardiology

This Research Topic includes six articles and one erratum on examples of new indications of nuclear medicine techniques in several fields of medicine, including oncology and cardiology.

Positron emission tomography (PET) is the most used nuclear medicine imaging method in oncology and Fluorine-18 fluorodeoxyglucose ([^18^F]FDG), which evaluates the glucose metabolism is currently the most common PET radiopharmaceutical. Beyond the well-known diagnostic role of [^18^F]FDG for staging, restaging and treatment response assessment of several tumors characterized by increased glucose metabolism, this method may also provide useful prognostic information. This is well underlined by the original article published by Nanni et al. on the prognostic value of hybrid morpho-functional imaging with PET/contrast-enhanced CT (PET/ceCT) performed before trans-arterial radioembolization (TARE) in patients with inoperable intrahepatic cholangiocarcinoma (iCCA). The authors reported a statistically significant correlation between some parameters at morpho-functional investigations at the baseline and overall survival and concluded that performing integrated pre-therapy imaging is critical for the prognostic stratification of patients with iCCA (Nanni et al.).

Remaining in the oncology field, Ouvrard et al. provided an overview on the current role of different nuclear medicine imaging techniques in evaluating bone metastases. The authors recognize that conventional bone scintigraphy using radiolabeled bisphosphonates is still widely used for the assessment of bone metastases (at diagnosis, during and after treatment), however, this method has some limitations and several nuclear medicine imaging methods including different radiopharmaceuticals have been developed for the assessment of bone metastases. The PET radiopharmaceuticals include [^18^F]NaF, the counterpart of bone scintigraphy, [^18^F]FDG for several tumors, radiolabeled choline and prostate specific membrane antigen (PSMA) ligands for prostate cancer, [^18^F]FDOPA and [^68^Ga]Ga-DOTA-peptides for neuroendocrine tumors. The authors describe the diagnostic performance of different nuclear imaging modalities in clinical practice for detecting and monitoring the therapeutic responses in bone metastases of diverse origins, addressing their limitations and implications for image interpretation (Ouvrard et al.).

There is currently a great effort in nuclear medicine to develop radiolabeled molecules that could be used for both imaging and therapy (theranostics) of different tumors. D'Onofrio et al. illustrate the promising literature data about the use of the Gastrin-Releasing Peptide Receptor (GRPR) as a target for the development of theranostic radioligands for luminal breast cancer with positive estrogen receptor expression. In the last decades, several GRPR-targeting molecules have been evaluated both at preclinical and clinical levels, however, most of the studies have been focused on prostate cancer. However, since luminal breast tumors account for about 80% of all breast cancer, many patients are likely to benefit from the development of GRPR-theranostics as summarized by the authors in their review (D'Onofrio et al.).

The COVID-19 pandemic had an impact on different medical fields including nuclear medicine. Minamimoto provides through a review a summary of the current state of oncology and cardiology PET examinations related to COVID-19, including preparation of the nuclear medicine department, trends in PET examinations, specific imaging findings on PET/CT with different tracers, imaging of complications of COVID-19, and the effects of COVID-19 vaccines on PET imaging findings (Minamimoto).

PET imaging with different tracers can be used for non-oncological diseases such as cardiovascular diseases. Dietz et al. reported an overview of arginine–glycine–aspartate (RGD)-PET agents in cardiovascular diseases. Literature showed an increasing role of RGD-based PET agents in patients with cardiovascular diseases. Overall, two main topics emerged: the infarcted myocardium and atherosclerosis both conditions characterized by increased RGD-based tracer uptake. Promising applications of RGD-based PET are emerging, such as prediction of remodeling processes in the infarcted myocardium or detection of active atherosclerosis, with potentially significant clinical impact (Dietz et al.; Frontiers Production Office).

Remaining in the cardiology field, Vançon et al. described a study protocol to investigate investigating cardiac transthyretin amyloidosis flow reserve before and after Tafamidis treatment. Patients with confirmed transthyretin related cardiomyopathy seen in the nuclear medicine departments of three large referral centers and treated with Tafamidis will be included. At baseline, patients will have a clinical and echocardiography evaluation. They will undergo a dynamic rest/stress cardiac scintigraphy with flow and reserve measurements before and 24 months after Tafamidis introduction. The primary outcome of this study will be the variation of stress and rest myocardial blood flow and flow reserve between baseline and 24 months after treatment. The effect of Tafamidis will be assessed by an intention to treat analysis (Vançon et al.).

In conclusion, the articles included in this Research Topic discuss different potential applications of hybrid imaging techniques in oncology, cardiology and inflammatory/infectious diseases demonstrating that several radiotracers will likely have a relevant role in the era of personalized medicine.

## Author contributions

DA: Supervision, Validation, Visualization, Writing – original draft, Writing – review & editing. GT: Conceptualization, Supervision, Validation, Visualization, Writing – original draft, Writing – review & editing.

